# Extended post-partum modern contraceptive utilization and associated factors among women in Arba Minch town, Southern Ethiopia

**DOI:** 10.1371/journal.pone.0265163

**Published:** 2022-03-16

**Authors:** Yibeltal Mesfin Yesgat, Tekilemariam Gultie Ketema, Samuel Abebe Dessalegn, Abraham Wallelign Bayabil, Muche Argaw Enyew, Eyaya Habte Dagnaw

**Affiliations:** 1 Department of Midwifery, College of Medicine and Health Sciences, Wolkite University, Wolkite, Ethiopia; 2 Department of Midwifery, College of Medicine and Health Sciences, Arba Minch University, Arba Minch, Ethiopia; 3 Department of Midwifery, College of Medicine and Health Sciences, Debre Tabor University, Debre Tabor, Ethiopia; University of Brescia: Universita degli Studi di Brescia, ITALY

## Abstract

**Introduction:**

Post-partum family planning is a novel strategy to reduce maternal and neonatal mortality by preventing unwanted pregnancy and unsafe abortion. However, little was done on community-based design to assess modern contraceptive use during an extended postpartum period in southern Ethiopia. Therefore, this study aimed to assess modern contraceptive use during extended postpartum period and factors associated among women who gave birth in the previous twelve months in southern Ethiopia.

**Methods:**

A community-based cross-sectional study was conducted among 416 women in Arba Minch town. A systematic random sampling technique was employed to select the enrolled women. Data were collected using a structured and pretested questionnaire. The data were entered into Epi-Data version 4.6 then exported to statically package of social science (SPSS) version 25 for data analysis.

**Result:**

Among enrolled postpartum women, 64.7% were used modern contraceptives for the last 12 months. Women were more likely to use a modern contraceptive during the extended period of postpartum if they resumed sexual intercourse (AOR:7.4 [4.08, 13.23]), received post-partum family planning counseling (AOR: 3.2 [1.95, 5.28]), and if they resumed menses (AOR: 5.3 [3.12, 9.15]) than the counterpart. Being young age women (AOR: 3.2 [1.05, 9.82]) compared to age above 35 years and married (AOR:3.2 [1.17–10.28]) compared to currently unmarried were significantly associated factors for modern contraceptive use during the extended period of postpartum.

**Conclusion:**

The level of modern contraceptive utilization during the extended postpartum period was satisfactory. Therefore, in light of this finding, there is a need to improve the strengthening and scale-up antenatal and postnatal counseling of contraceptive use during the extended postpartum period, advice on preceding the return of menses, and give better attention for older age and unmarried women education on family planning.

## Introduction

Modern contraceptive use during the extended postpartum period is the utilization of any modern methods in one-year follow-up after childbirth. It is the critical period for the prevention of unwanted and closed species pregnancy [1]. Globally, over 90% of women want to either delay or avoid pregnancy during the twelve- months of the postpartum period. This percent increase to 95% in low and middle-income countries but, 70% of women do not use contraceptives [2, 3].

Most countries in sub-Saharan African are characterized by high population growth. Until recently, women have 5.1 children by average in a lifetime. The main driving force for high fertility is low access to healthcare including family planning services and a negative attitude to family planning(FP) due to traditional beliefs and religion. This high fertility rate is reduced by providing contraceptives for women, especially on extended postpartum periods.

Postpartum family planning(PPFP) reduce high fertility and increase maternal and child health by preventing unintended short interpregnancy interval and unsafe abortion [4]. Short interpregnancy interval associated with increased maternal morbidity such as bleeding, premature rupture of membrane, anemia, and sepsis. In addition to health, it has also economic and social negative impacts on the community. The large family size is attendant to poor education, low standard of living, and inability to fulfill one’s dream [5–7]. So. This serious problem is avoided by providing effective family planning with an extended postpartum period.

The government of Ethiopia is working intensively to ensure affordable and accessible contraceptive methods. The country had written the Health Sector Transformation Plan to reach additional 6.2 million women and increase contraceptive prevalence to 55% until 2020. However, the Ethiopian demographic health survey (EDHS) 2016 reported that 1in 5 married women had an unmet need for FP [8–10]. The uptake of PPFP during the extended period in Ethiopia ranges from 10.3% to 80.3% [11, 12]. The cross-sectional studies in Aksum and Gondar town showed that 48% and 45.8% of women started using modern contraceptives during the extended postpartum period respectively [13, 14].

Women menses and sexual intercourse resumption, unable to know the return of fecundity, educational level and receive PPFP counseling are some factors affecting extended period PPFP use [11, 13, 15, 16].

Even though various institutional-based studies were conducted in Ethiopia to assess modern contraceptive use and associated factors during the extended postpartum period, little was done with community-based design on postpartum modern contraceptive use during an extended postpartum period in the context of southern Ethiopia. Therefore, this study aimed to assess extended postpartum period modern contraceptives use and associated factors among women delivered for the last twelve months in Arba Minch town, southern Ethiopia.

## Methods and materials

### Study area

The study was carried out in Arba Minch town which is found in the southern part of Ethiopia. It is located in the Gamo zone, southern nation nationalities peoples region about 505 km south of Addis Ababa. The town has 11 kebeles with a population of approximately 112,724 which are 56,137 males and 56,587 females. There is one General Hospital, three public health centers, eleven health posts providing maternal and child health services, including family planning and immunization.

Study period and design. A community-based crossectional study was conducted from 1 Oct to 30 Nov 2019.

### Study participants

The source populations encompassed all reproductive age (15–19) postpartum women who gave birth for the last 12 months before this study. Women who gave birth in the last 12 months and lived in the study area at least for six months preceding the survey regardless of their birth outcome were included in the study and women who were critically ill were excluded.

### Sample size determination and procedure

The sample size was calculated using the single population proportion formula; 95% confidence level, 5% margin of error and considering 48% the prevalence of postpartum modern contraceptive in Aksum town [13].

n = (Zα/2)2 P (1—P)

        d^2^

    n = (1.96)2x 0.48x0.52 = 384

        (0.05)^2^

      Where; p = prevalence of postpartum modern family planning (48%)

    d = marginal error between the samples and population (0.05)2

    Zα/2 = critical value at 95% certainty (1.96)2

      n = calculated sample size = 384

And adding a 10% non-response rate, the final sample size become = **423**

Out of a total of 11 kebeles, 4 Keble were selected randomly. The eligible study participant’s household was drawn from vital registration by a health extension worker. The total sample size (n = 423) size was allocated by using proportional to the total number of postpartum women in the selected Kebeles (Chamo (88), Wuha Minch(97), Kulfo (114), and E/ber (125)). Then, systematic random sampling was applied to selected postpartum women in every Kth (3rd) interval of the eligible household. In a case when the study participants are not able to interview for some reason, an attempt is made two times to interview the respondent and after all, the next study participant was asked. If there were more than one eligible woman in one household, one participant was selected randomly.

### Data collection instrument and procedure

The data were collected by using a pre-tested interview administrated structured questionnaire. The questionnaire consists of four parts such as part socioeconomic and demographic characteristics, reproductive history and maternal health care, knowledge, and utilization of modern contraceptives. The collected information regarding knowledge of modern contraceptive methods was after describing each method and asking respondents if they had heard of it. The data collection tool (questionnaire) was prepared first in English and then translated to Amharic, which was translated back to English to ensure consistency. Two diploma midwives, two clinical nurses, and one BSc Midwife supervisor collected data. The interviewers were trained for one day before the actual data collection on interviewing approach and data recording.

### Data processing and analysis

All filed questioners were cheek for completeness consistency and accuracy, and then the data were entered into Epi-Data version 4.6 then exported to statically package of social science (SPSS) version 25 for data analysis. Descriptive statistics such as percentages, frequencies were used to characterize the study population as appropriate. Bivariate analysis was computed to identify potential variables for the multivariable logistic regression model. Variables with *p*-value of ≤0.25 in the bivariable analysis have been considered for multivariable analysis. Adjusted-odds ratio along with 95% confidence interval (CI) was used to measure association in the multivariable logistic regression model. The fitness of the model was checked by the Hosmer-Lemeshow goodness of fit test not statically significant p-value 0.183. Finally, *p*-value <0.05 was considered as a cut-off point to declare a significant statistical association.

### Ethical clearance and consent to participate

Ethical clearance was obtained from the institutional research ethical review board of Arba Minch University College of Medicine and Health Science. A permission letter was written to Arba Minch town health office administration to conduct the study and the other permission letter was obtained from the Arba Minch town administration. After the purpose and objective of the study were informed verbal and written consent was obtained from each study participant. Finally, parental informed consent was taken for those ages less than 18 years.

## Result

### Socio-demographic characteristics of the respondent

In this study, 416 postpartum women participated with a response rate of 98.3%. The age of women ranges from 16–48, with a mean age of 28.2 (SD ±4. 96) years. The majority of women (94.2%) were married. About 234 (56.2%) women menses resumed. The majority of the respondents 345 said they have a plan for future pregnancy. Only 168 and 126 respondents have four or more ANC visits and immediate postnatal follow up respectively. About 242 (58.2%) women had family planning counseling during ANC and PNC (**Table 1**).

**Table 1:** Frequency distribution of postpartum women by their socioeconomic and demographic characteristics in Arba Minch town, 2019 (N = 416).

Variable	Number(n)	Percent (%)
Age		
15–24	92	22.2
25–34	276	66.3
≥35	48	11.5
Marital status		
Married	392	94.3
Currently unmarried	24	5.7
Women education		
No formal education	67	16.1
Primary	122	29.3
Secondary	110	26.4
Diploma and above	117	28.2
Partner education(N = 392)		
No formal education	26	6.6
Primary	77	19.7
Secondary	133	33.9
Diploma and above	156	37.8
Ethnicity		
Gamo	229	55.1
Gofa	37	8.9
Wolayita	35	8.4
Amhara	68	16.3
Oromo	25	6.0
Others*	22	5.3
Occupation		
Housewife	105	25.2
Self-employed	110	26.4
Government Employed	103	24.8
Merchant	54	13.0
Others**	44	10.6
Religion		
Protestant	193	46.4
Orthodox	191	45.9
Others***	32	7.7
Monthly income		
≤1000	85	20.4
1001–2000	81	19.5
2001–3000	99	23.8
>3000	151	36.3
Resumed Menses		
Yes	234	56.2
No	182	43.8
Resumed Sexual Intercourse		
Yes	314	75.5
No	102	24.5
Future Fertility Desire		
Yes	343	82.5
No	73	17.5
Postpartum period(wk.)		
0–12	109	26.2
13–26	173	41.6
27–38	97	23.3
39–50	37	8.9
ANC Follow Up		
No ANC follow up	30	7.2
<4 Visits	21	52.4
4 and above visits	168	40.4
PNC Follow Up		
Yes	126	30.3
No	290	69.7
FP Counseling During ANC and PNC		
Yes	242	58.2
No	174	41.8

Other

*: Gurage, Tigre, Konso; Others

** = Daily laborer, Unemployed; Others

***: Muslim, Catholic, FP: Family planning, ANC: Antenatal care, PNC: Postnatal care

### Knowledge, attitude, and media exposure about modern contraceptive

Knowledge of at least one modern contraceptive likely to have about it is 98.1%. Concerning the attitudes of the respondents towards the benefits of modern contraceptive utilization about half (50.7%) of them had positive attitudes. One hundred eighty-three (44.0%) postpartum women had got information about family planning from the media.

### Modern contraceptive use in the extended postpartum period

The prevalence of extended postpartum modern contraceptive use was 64.7% [95% CI: (59.6, 69.2)]. Injectable contraceptives 137 (50.9%) and implant 77 (28.6%) were the most frequently used methods.

### Factors associated with postpartum modern contraceptive use

In the multivariable logistic regression analysis, resumed sexual intercourse ((AOR: 7.4 [4.08, 13.23]), received post-partum family planning counseling (AOR: 3.2 [1.95, 5.28]) and resumption of menses (AOR: 5.3 [3.12, 9.15]) than the counterpart. Being young age women (AOR: 3.2 [1.05, 9.82]) compared to age above 35 years and married (AOR = 3.2 [1.17–10.28]) compared to currently unmarried were significantly associated with modern contraceptive use during the extended postpartum period (**Table 2**).

**Table 2:** Factors associated with modern contraceptive use during the extended postpartum period, Arba Minch town, 2019 (n = 416).

Variable	PPFP use		COR(95%CI)	AOR	P value
				(95%CI	
	Yes	No			
Age of the women					
15–24	58	34	2.39(1.17–4.87)	4.2(1.66–10.64)	0.002
25–34	191	85	3.15(1.68–5.89)	3.2(1.05–9.82)	0.004
≥35	20	28	1.00	1.00	
Marital status					
Married	261	131	3.9(1.66–9.55)	3.2(1.17–10.28)	0.025
Currently unmarried	8	16	1.00	1.00	
Women Education					
Diploma and above	85	32	2.3(1.219-	1.2(0.52–2.99)	0.065
Secondary	72	38	4.291)	0.8(0.35–1.88)	0.16
Primarily	76	46	1.6(.87–3.31)	0.9(0.43–2.14)	0.08
No formal education	36	31	1.7(.78–3.60) 1.00	1.00	
ANC Visits					
4 and above	125	43	4.4(1.94–9.78)	2.4(0.85–6.72)	0.48
Less than 4 visits	132	89	2.3(1.12–5.19)	1.6(0.51–5.14)	0.09
No visit	12	18	1.00	1.00	
Media Exposure					
Yes	140	43	2.6(1.71–4.18)	1.3(0.75–2.38)	0.32
No	129	104	1.00	1.00	
PPFP Counseling					
Yes	188	54	3.9(2.61–6.12)	3.2(1.95–5.28)	0.001
No	81	93	1.00	1.00	
Knowledge of PPFP					
Yes	267	141	5.7(1.132–28.513)	1.8(0.28–12.06)	0.56
No	2	6	1.00	1.00	
Menses Resumed					
Yes	185	49	4.4(2.87–6.68)	5.3(3.12–9.15)	0.0001
No	84	98	1.00	1.00	
Sexual Intercourse resumed					
Yes	237	77	6.7(4.12–11.11)	7.4(4.08–13.23)	0.0001
No	32	70	1.00	1.00	

## Discussion

This community-based design research aimed to identify the use of modern contraceptives and associated factors in Arba Minch town among women in the extended postpartum period. In this study, about 64.7 percent of women use modern contraceptives in twelve months of the postpartum period. The finding is in line with the study done in northwest Ethiopia (63%) [17].

This finding was higher than studies done in the Axsum town, northern Ethiopia (48%), Tanzania (11.6%), Uganda (28%), and Ghana (26.3%) [13, 18–20]. The possible explanation for this variation may be due to differences in socio-demography, study design, and year of study. The Axsum town study was conducted in 2015 and increased access and awareness of contraceptives in past five years.

Inversely, this finding is lower than Addis Ababa (80.3%) and Kenya (86.3%) findings [12, 20]. The possible justifications might be variations in study design and setting. The study conducted in Addis Ababa include postpartum women until 24 months. Addis Ababa may also have better health care facilities and access for FP the than study area.

This finding showed that young age post-partum women were almost four times use modern contraceptives than the age of 35 years or more. This agrees with the study in Gondar town, North West Ethiopia, Uganda, Malawi, and Ghana [13, 19–21]. This could be due to older age women’s less frequency of sexual desire than younger women, declared in fecund after 40 and less ideal children desire. So, this is an alarm for FP providers to address all age postpartum women to improve modern contraceptive use.

The finding of this study revealed that the marital status of postpartum women is significantly associated with modern contraceptive use. This study indicates married postpartum women were 3.2 times more likely to utilize modern contraceptives than currently unmarried. This finding was supported by the study done at Debre Tabor, Addis Ababa, and Kenya [17, 22, 23]. The finding indicates that married postpartum women might early begin sexual intercourse and the couple may discuss FP utilization for a plan for the next birth.

This finding also revealed that women counseled about FP during ANC and PNC significantly associated PPFP utilization. This study indicates women who received FP counseling during ANC and PNC were more likely to use modern contraceptives than not counseled. The result is supported by Axum, Gondar town, and Malawi[13, 24]. The possible justification could be increasing women’s awareness about contraceptives motivated them to use FP counseling. Due to this, the ANC and PNC providers better give more attention to FP counseling to enhance extended PPFP utilization.

Mothers whose menses resumed were almost five times more likely to utilize modern contraceptives. This is inconsistent with studies in Addis Ababa, Gondar, Northwest Ethiopia, Gozamen District, East Gojjam Zone, Northwest Ethiopia [21, 22, 25]. This is because postpartum women may be aware of their fertility returning when menses return and could be due to postpartum women believing that a return of fertility only occurs along with the return of menses. Therefore, it is better to educate women may ovulate before the return of first menses after childbirth.

The other factor associated with PPFP utilization is sexual intercourse resumption. Women who have sexual intercourse resumed 7.4 times more likely to use modern contraceptives compared with not started sexual intercourse. This finding is in concord with studies done in Hosanna and Axsum [13, 15]. This might be due to women’s resumption of sexual activity fear to get pregnant.

Finally, this study has some limitations and strengths to be noted. The study tried to address the determinant factor of PPFP utilization with a community-based design important for generalization at the community level. The limitation of this study is the design. A cross-sectional study made it impossible for a causal relationship. Another limitation of this study is recall bias.

## Conclusion

In this study, the level of PPFP utilization during an extended period of postpartum was satisfactory. Therefore, in light of this finding, there is a need to improve PPFP utilization by strengthening and scale-up of intervention geared towards these factors, antenatal and postnatal counseling of postpartum family planning, advice on preceding the return of menses, and to give better attention for older age and unmarried women education on family planning.

## Supporting information

S1 File Amharic and english questioner. (DOCX)

S2 File Dataset. (SAV)

## List of abbreviation

ANC: Antenatal Care
EDHS: Ethiopia Demographic Health Survey
PPFP: Postpartum Family Planning
FP: Family Planning
PNC: Postnatal Care
SDG: Sustainable Development Goals
WHO: World Health Organization
