# Supplementary material for: Extended post-partum modern contraceptive utilization and associated factors among women in Arba Minch town, Southern Ethiopia
Source: PLoS One. 2022 Mar 16;17(3):e0265163. doi: 10.1371/journal.pone.0265163 (PMC8926256; doi:10.1371/journal.pone.0265163)
Supplement: S1 File — (DOCX) [file pone.0265163.s001.docx]

**Annex II: English questionnaire**

**Arba Minch University, College of Medicine and Health Sciences, Department of Midwifery**

**Consent form**

Good morning! / Good afternoon! My name is --------------. I am member of a research team for master’s thesis project in Arba Minch University, College of Medicine and health science, department of Midwifery. We are conducting a research to assess utilization and associated factors of modern contraceptive during extended postpartum period among women who Gave Birth in the last 12 months in Arba Minch Town. The study is directly related to women who give birth in the last 12 month, you may be one of the women who will be selected to participate in this study. Therefore you are kindly requested to participate in this study and provide the information required from you. Your participation in this study is completely on voluntary basis and you have the right to refuse from participating. Your responses will be kept confidential and there will be no way of linking your individual responses to the final results of the study findings. We would like to inform you that the responses that you provide to the questions are very essential for the successful accomplishment of this study.

Are you willing to participate in this study to give your responses based on the questionnaire?

Yes ----------signature--------------------------------------

No

Name and Signature of the data collector _____________________

Name and signature of the supervisor ________________________

Date of interview _______________________________________

Code ______________________________________

Assent form

I know that I can choose to be in the research study or choose not to be in the research study. I know that I can stop whenever I want. I have read this information (or had the information read to me) and I understand it. I have had my questions answered and know that I can ask questions later if I have them. I understand any changes to this will be discussed with me. (If illiterate: women who are illiterate should be included their thumb print as well).

Signature of Client: __________ Date: ________

Witness: I have witnessed the accurate reading of the assent form to the potential participant, and the individual has had the opportunity to ask questions. I confirm that the individual has given assent freely (literate witness must sign (if possible, this person should be selected by the participant, not be a parent, and should have no connection to the research team).

Signature of witness ________Date ________

Researcher confirms it: I have accurately read or witnessed the accurate reading of the assent form to the potential participant, and the individual has had the opportunity to ask questions. I confirm that the individual has given assent freely.

Name of data collector _________________ Signature of data collector_____ __Date_______

| **Part I:- socio-demographic characteristics** | | | | | | | | | |
| --- | --- | --- | --- | --- | --- | --- | --- | --- | --- |
| No | Questions | | Responses | | | Skip | | | |
| 101 | How old are you? | | ------------years old | | |  | | | |
| 102 | What is your religion? | | 1. 1.Orthodox 2. 2.Muslim 3. 3.Protestant 4. 4.Catholic 5. 5.Others specify------ | | |  | | | |
| 103 | To which Ethnic group do you belong? | | 1. 1.Gamo 2. 2.Goffa 3. 3.Wolayita 4. 4.Amhara 5. 5.Oromo 6. 6.Others, specify------------ | | |  | | | |
| 104 | What is your level of education? | | 1. 1. Unable to read and write 2. 2 .Read and write 3. 3. 1-8^th^ class 4. 4. 9-12^th^ class 5. 5, Diploma and above | | |  | | | |
| 105 | What is your occupation? | | 1. 1. Housewife 2. 2. Self-employees 3. 3. Government employees 4. 4. Merchant 5. 5. Labor work 6. 6. Farming 7. 7.Student 8. 8.Job Less 9. 9. Others, specify------------ | | |  | | | |
| 106 | What is your marital status? | | 1. 1. Single 2. 2. Married 3. 3. Divorced 4. 4.Widowed | | |  | | | |
| 107 | If Q 106 married what is your husband’s occupation? | | 1. 1. Government employees 2. 2. Merchant 3. 3. Labor work 4. 4. Student 5. 5. Self-employees 6. 6. Job less 7. 7. Farming 8. 8. Others, specify------------ | | | For Married only | | | |
| 108 | If Q 106, married what is your husband’s education status? | | 1. 1. Unable to read and write 2. 2. Read and write 3. 3. 1-8^th^ class 4. 4. 9-12^th^ class 5. 5. Diploma and above | | |  |  |  |  |
| 109 | How much birr is your monthly income? | | Birr/month------------------------ | | |  | | | |
| **Part-II: Knowledge of postpartum modern family planning (PPFP) services** | | | | | | | | | |
| No | Question (**did you know**?) | | | | | 1. Yes | | | No |
| 201 | The pill (oral contraceptives) - prevents the release of egg from the ovaries which is taken every day at the same time | | | | |  | | |  |
| 202 | IUD(loop) –it’s a small flexible family planning method  inserted in the uterus that’s prevents fertilization | | | | |  | | |  |
| 203 | Male condom-method that covers erected pines which is  worn before sex that prevents pregnancy and STDs | | | | |  | | |  |
| 204 | Female condom-it’s a method produced from plastic which is inserted inside the vagina before sexual intercourse | | | | |  | | |  |
| 205 | Implants-have a size of match stick inserted under the arm skin that prevents fertilization by controlling the female hormone? | | | | |  | | |  |
| 206 | Injectable-it’s a method given on arm or thigh every three month that prevents release of egg from ovaries. | | | | |  | | |  |
| 207 | Emergency hormonal contraception-is a method used when a women forgets to take her pills/injectable when she has an unprotected sex including rape | | | | |  | | |  |
| 208 | Tubal ligation-its tying the fallopian tube that prevents  fertilization using simple surgery | | | | |  | | |  |
| 209 | Vasectomy-it’s a method used to prevent the passing of sperm through vas difference using simple surgery | | | | |  | | |  |
| 210 | Breast feeding –protects pregnancy for 6 months if mother gives only breast milk for neonate and menses doesn’t resumed | | | | |  | | |  |
| **Attitudes of postpartum modern family planning (PPFP) services** | | | | | | | | | |
| **No** | | Question /Items | | **Responses** | | | | | |
|  |  |  |  | **Agree** | | **Not sure** | | **Not Agree** | |
| 212 | | Children will have better opportunities for education, If their parents practice FP. | |  | |  | |  | |
| **213** | | FP will help improve one’s standard of living | |  | |  | |  | |
| **214** | | FP helps a mother to regain strength before her next baby | |  | |  | |  | |
| **215** | | A woman who has too many children looks tired and wears out. | |  | |  | |  | |
| **216** | | Child spacing does not helps to protect the health of children and mothers. | |  | |  | |  | |
| **217** | | FP causes a loss of confidence between a husband and a wife | |  | |  | |  | |
| **218** | | Wives who practice FP will be abandoned by their husbands | |  | |  | |  | |
| **219** | | A couple that practice FP will have conflict in their marriage | |  | |  | |  | |
| **220** | | Contraceptive use may cause infertility in a Woman | |  | |  | |  | |
| **221** | | A couple that practice FP has a happy family | |  | |  | |  | |
| **Part lll: Male involvement in postpartum modern contraceptive utilization** | | | | | | | | | |
| 301 | Do you discuss with your partner about modern postpartum contraceptive? | | | | 1. 1.Yes 2. 2. No | | | |  |
| 302 | Does your partner support you when you want to use modern postpartum contraceptive. | | | | 1. 1. Yes 2. 2. No | | | |  |
| 303 | From where/whom did you get the information about modern postpartum contraceptive.  More than one answer is possible | | | | Health care professionals-------------------1  Friends -----------------------------------------2  Media ------------------------------------------3 Others (specify) ---------------------------------4 | | | |  |
| **Part IV:-Reproductive health history and maternal health services** | | | | | | | | | |
| 401 | Was it your first delivery? | | | | 1. 1.Yes 2. 2. No | |  | | |
| 402 | How many live children do you have | | | | ------------ | |  | | |
| 403 | Did you have a history of abortion | | | | 1. 1. Yes 2. 2. No | |  | | |
| 404 | What was the status of your last pregnancy? | | | | 1. 1. Planned 2. 2. Unplanned | |  | | |
| 405 | Did you have antenatal care for your recent pregnancy? | | | | 1. 1. Yes 2. 2. No | | If no, skip Q 409 | | |
| 406 | If Q “406” yes How many times did you attend the antenatal clinic? | | | | 1. 1. One visit 2. 2. Two visit 3. 3. Three visit 4. 4. Four and more visits | |  | | |
| 407 | Did you get counselling about family planning during pregnancy? | | | | 1. 1. Yes 2. 2. No | |  | | |
| 408 | Where was the place of delivery? | | | | 1. 1. Government Hospital 2. 2. Health center 3. 3. Health Post 4. 4. Privet clinic 5. 5. Privet Hospital 6. 6. NGO health institute 7. 7. At Home 8. 8. Others (specify) -------------- | |  | | |
| 409 | Who assisted you with the delivery? Record all mentioned | | | | Doctor-------------------------1 Nurse/midwife --------------2  Relatives …… …… ……..3  Untrained traditional attendant…4  Trained traditional attendant….5  Other ----------------------------------6 | |  |  |  |
| 410 | Did you have postnatal care visit? | | | | 1. 1.Yes 2. 2. No | | If no, skip to Q 412 | | |
| 411 | If Q “410” yes did you receive counselling about Family planning | | | | 1. 1. Yes 2. 2. No | |  | | |
| 412 | Do you want to have children in the future? | | | | 1. 1. Yes 2. 2. No | | If no, skip to Q 414 | | |
| 413 | When would you like to have the next child? | | | | 1. 1. After one year 2. 2. After two years 3. 3. After three years 4. 4. After four years and above | |  | | |
| 414 | Is your menses resumed after your recent childbirth | | | | 1. Yes  2. No | | If no, skip to Q 416 | | |
| 415 | \| If Q “418” yes, when menses resumed after birth? \|  \| \| --- \| --- \| | | | | -------------------weeks | |  | | |
| 416 | Have you resumed sexual activity since the birth of your child? | | | | 1. 1. Yes 2. 2. No | | If no, skip to  Q 418 | | |
| 417 | If Q “416” yes, At what age of your child did you resume sexual intercourse? | | | | 1. 1. Before 6week 2. 2. 6 weeks - 3 months 3. 3. 4 - 6 months 4. 4. 7 - 9 months 5. 5. 10 - 12 months | |  | | |
| 418 | What is the age of your infant currently? | | | | 1. ……………in Weeks | |  | | |
| **Part V:-About practice of modern contraceptives in the extended postpartum period** | | | | | | | | | |
| 501 | Did you use modern contraceptive method within 12 months after delivery? | | 1. 1.Yes 2. 2. No | | | | No. Skip to  505 | | |
| 502 | If Q “501” yes, when did you start using modern family planning method after delivery? | | ------------------weeks after birth | | | |  | | |
| 503 | If Q “501” yes, what method did you use? | | 1. 1. Pill 2. 2. IUCD 3. 3. Injectable 4. 4. Male condom 5. 5. Implant 6. 6. Female Sterilization 7. 7. Breast Feeding for 6 months 8. 8. Emergency pill 9. 9. Others(specify)-------------- | | | |  | | |
| 504 | From where did you get the FP method? | | 1. 1. Government health facility 2. 2. Private health facility 3. 3. NGO facility 4. 4. Pharmacies/drug venders 5. 5. Others(specify----------------- | | | |  | | |
| 505 | If no ‘Q’ 501 Why don’t you want to use family planning after delivery? | | 1. 1.Fear of side effects 2. 2.Want to deliver soon 3. 3.Fear of change in breast milk 4. 4. Feeling of not at risk of pregnancy due to amenorrhea 5. 5. Husband/partner opposed 6. 6. Not married 7. 7.Plan to abstain/avoid sex 8. 8. Don’t know FP methods 9. 9. Others (specify)………….. | | | |  | | |

**ANNEX V ; QUESTIONNAIRES (AMHARIC VERSION)**

**አማርኛ መጠይቅ**

**የመረጃ ፎርም**

እንደምን አደርሽ/ ዋልሽ? እኔ ሰሜ --------------------------------- ነዉ፡፡ የመጣሁት ከአ/ም ዩኒቨርሲቲ የጤና ሳይንስ ኮሌጅ ሚድዋይፈሪ ት/ክ ነዉ፡፡ ከወለድሽ በሁዋላ በ12 ወራት ውስጥ ስለተጠቀምሽው የቤተሰብ ምጣኔ አንዲንድ ጥያቄዎችን ልጠይቅሽ ነው፡፡ ከአንቺ የምናገኘው ምላሽ የቤተሰብ ምጣኔ አገሌግልትን ለማሻሻል ለሚመለከተው አካል ለመጠቆም ይረዲናል፡፡ በቃለ መጠይቁ እንዴትሳተፊ የተመረጥሽው እድል ስለደረሰሽ ነው፡፡ ስለ ጥናቱ አጠቃሊይ መረጃ እንዲኖረሽ ከዚህ በታች ያለው መረጃ ይጠቅምሻሌ፡፡ የጥናቱ ርእስ የቤተሰብ ምጣኔ ተጠቃሚነት በ 12 ወራት ውስጥ ከወሉዴ በሁዋሊ እንዱሁም በተጠቃሚነት ሊይ ሌዩነት የሚያመጡ ምክንያቶች፡፡

መግቢያ ከወሉዴ በሁዋሊ ባለ 12 ተከታታይ ውራት ውስጥ እርግዝና እንዲይከሰት የቤተሰብ ምጣኔን መጠቀም ያስፈሌጋሌ አንዲት እናት ከወለደች በሁዋላ ከሁለት አመት ባነሰ ጊዜ ውስጥ ካረገዘች እስዋም ሆነች ሌጅዋ አደጋ ሊይ ይወዴቃሉ፡፡ ነገር ግን በዚህ ጊዜ ውስጥ የቤተሰብ ምጣኔ መጠቀም የእናቶችንና የህጻናትን ሞት ይቀንሳሌ፡፡

የጥናቱ አላማ ከወሊድ በሁዋላ በ 12 ወራት ውስጥ የቤተሰብ ምጣኔ ተጠቃሚነት መጠንን ለመለካት እና በተጠቃሚነት ሊይ ሌዩነት የሚያመጡ ምክንያቶችን ለማወቅ

የጥናቱ ጥቅም ጥናቱ ለተሳታፊዋ ፈጣንና ቀጥተኛ ጥቅም ባይኖረውም የቤተሰብ ምጣኔ አገሌግልትን ለማሻሻሌ ለሚመለከተው አካል ለመጠቆም ይረዲናሌ፡፡

በጥናቱ የሚመጣ ችግር ይህ ጥናት በተሳታፊዋ ሊይ ምንም አይነት ችግር አይኖረውም

የተሳታፊዋ መብት ተሳትፎሽ ባንቺ ፍቃደኝነት ሊይ የተመሰረተ ነው፡፡ ይህ ቃለ መጠይቅ ከ 15-20 ደቂቃ የፈጃል፡፡ በጥናቱ ነጻነት ካልተሰማሽ በማንኛውም ጊዜ ማቁዋረጥ ትችያሇሽ፡፡ በዚህ መጠይቅ ሊይ ስምሽ አያሥፈሌግም፡፡

**የስምምነት ቅጽ**

የተጠያቂ ቃል

የኔን በዚ ጥናት መሳተፍ በተመለከተ ከላይ የቀረበልኝ መረጃ ግልጽ ሆኖልኛል፡፡ የጥናቱ አላማ በምረደው ቋንቋ ተገልጦልኛል፡፡ ከዚህም በተጨማሪ ጥያቄ እንድጠይቅ አጋጣሚ የተሰጠኝ ከመሆኑም በላይ ጥያቄዎች በተገቢው መንገድ መልስ አገኝተዋል፡፡

የኔ በዚህ ጥናት መሳተፌ ሙለ በፍቃደኝነት ላይ የተመሰረተ ነው፡፡እኔ የምሰጠው መረጃ በሚስጥር እንደሚያዝና በፈለኩበት ሰአት ቃለምልልሱን ማቆም እንደምችል ገብቶኛል፡፡ የኔ በዚ ጥናት መሳተፌም ሆነ አለመሳተፌ ከዚህም ሆነ ከሌላ ተቋም በማገኘው አገሌግልት ላይ ምንም ተጽዕኖ እንደማይነኖረው ተገንዝቤያለሁ፡

ፉርማ / ምልክት ………………………………………

የጠያቂ ቃል

እኔ ከዚ በታች ስሜ የተጠቀሰው ለፍቃደኛ ተሳታፉዋ በምትረዳው ቋንቋ በጥናቱ ውስጥ ያለትን ስርዐቶች እና ደንቦች እንዱሁም ጥቅሞቹን አስረድቻለሁ፡፡

የጠያቂ ስም………………………ፊርማ……………………ቀን…………

**ክፍል 1 የማህበራዊ ኢኮኖሚያዊ እና ስነህዝባዊ ባህሪያት**

| ቁጥር | ጥያቄ | መልስ |  |
| --- | --- | --- | --- |
| 101 | እድሜ ስንት ነዉ | ……….. |  |
| 102 | ሀይማኖት | 1. ኦርቶድክስ ክርስቲያን  2. ፐሮቴሰታንት  3. ሙስሊም  4. ካቶሉክ  5. ሌላ |  |
| 103 | ብሄር | 1. ጋሞ  2. ጎፋ  3. ወላይታ  3. አማራ  4. ኦሮሞ  5. ሌላ ይጠቀስ-------------------- |  |
| 104 | የትምህርት ደረጃ | 1.ማንበብም መጻፍም እችላለሁ  2. ማንበብም መጻፍም አልችልም  3. የመጀመሪያ ደረጃ (1-8)  4. ሁለተኛ ዯረጃ (9-12)  4. የቴክኒክና ሙያ  5. የከፍተኛ ደረጃ |  |
| 105 | ዋና ስራሽ ምንዴን ነው? | 1. የቤት እመቤት  2. ንግዴ/ ተያያዝነት ያለው ስራ  3. የመንግስት ተቀጣሪ  4. የቀን ሰራተኛ  5. ተማሪ  6.የግል ስራ  7. ስራ አጥ  9. ሌላ ይገለጽ--------- |  |
| 106 | አሁን ያለሽበት የጋብቻ ሁኔታ ምን ይመስላል? | 1. አይ አሁን አላገባሁም  2. በህጋዊ አግብቻለሁ/ አበሬ እየኖርኩኝ ነው( የሰማኒያ ሚሰት)  3. ተፋትቻለሁ  4. ባልቤቴ ሞቶብኛሌ  7. ሌላ ካለ ይገለፅ.. | If No, skip Q 109 |
| 107 | የባልሽ ስራ ምን ነዉ | 1.የመንግስት ተቀጣሪ  2.ነጋዴ  3. የቀን ሰራተኛ  4. ተማሪ  5.የግል ስራ  6. ስራ አጥ  7. ሌላ ይገለጽ--------- |  |
| 108 | የባልሽ የትምህርት ደረጃ | 1.ማንበብም መጻፍም ይችላል  2. ማንበብም መጻፍም አይችልም  3. የመጀመሪያ ደረጃ (1-8)  4. ሁለተኛ ዯረጃ (9-12)  4. የቴክኒክና ሙያ  5. የከፍተኛ ደረጃ | |
| 109 | የወር ገቢሽ ስንት ነዉ | ብር በወር…….. | |

| **ክፍሌ 2 ስለ ዴህረ ወሉዴ የቤተሰብ ምጣኔ ያለሽ እውቀት እና ውይይት ላይ ጥያቄዎችን ልጠይቅሽ ነው፡፡** |
| --- |

|  | **የእርግዝና መከላከያ ዘዴ** | መልስ | |
| --- | --- | --- | --- |
|  |  | አዎ | የለም |
| 201 | **እንክብል(የሚዋጡ**)-በየቀኑ ተመሳሳይ በሆነ ሰአት የሚወሰድ እና እንቁላል ከእንቁልጤ እንዲይለቅ በማድረግ እርግዝናን የመከላከያ ዘዴ ነው |  |  |
| 202 | **በማህፀን ውስጥ የሚቀመጥ (ለፕ)**-አነስተኛ እና ተጣጣፉ በማህጸን ውስጥ የሚቀመጥ የእርግዝና መከሊከያ ዘዴ ሲሆን የወንዴ የዘር ፍሬ ከእንቁላል ጋር ዕንዳይገናኝ በማገድ እርግዝናን ይከላከላል |  |  |
| 203 | የወንድ ኮንድም-የቆመ የወንድ ብልትን የሚሸፍንና ከግብረ ስጋ  ግንኙነት በፉት የሚጠለቅ ቀጭን ላቴክስ ከተባለ ጎማ መሰል ቁስ  የተሰራ የእርግዝና እና የአባላዘር በሽታ መከላከያ ዘዴ ነው |  |  |
| 204 | ሴት ኮንድም- ከግብረ ስጋ ግንኙነት በፉት የሴቷ ብልት ውስጥ የሚገባ ከፕላስቲክ የተሰራ የእርግዝና መከላክያ ዘዴ ነው |  |  |
| 205 | **በክንዴ ቆዲ ሰር የሚቀበር** (ኢምፕሊንት)-የክብሪት እንጨት የሚያክል በክንዴ ቆዳ ስር የሚቀበር የእርግዝና መከላከያ ሲሆን በሴትዋ ቅመም(ሆርሞን) ለይ ተጽእኖ በማድረግ ውህደት እንዲይፈጠር ያደርጋል |  |  |
| 206 | **መርፌ**-በላይኛው ክንድ ወይም በታፊ በየሶስት ወሩ የሚሰጥ እንቁላሌ ከእንቁላል እጢ እንዳይወጣ የሚያደረግ እርግዝናን የመከላከያ ዘዴ |  |  |
| 207 | **የድንገተኛ እርግዝና መከላከያ**-አንዱት ሴት ኪኒን/መርፋ መወጋት  ስትረሳ ወይም መደፈርን ጨምሮ ያለ መከላከያ ጥንቃቄ የጎደለው  የግብረ ስጋ ግንኙነት ስታደርግ የሚወሰድ ኪኒን ነው |  |  |
| 208 | **የእንቁላል ማስተላለፉያ ቱቦን ማስቋጠር** (ቲዩባሌ ሉጌሽን)-የሴትዋን የእንቁላል ማስተላለፉያ ቱቦ ከወንዴ የዘር ፍሬ ጋር እንዳይገናኝ የሚያደርግ በቀላል የቀድ ጥገና የሚሰራ የእርግዝና መከሊከያ ዘዴ ነው |  |  |
| 209 | የወንዴ የዘር ማስተላለፍያ ቱቦን መቋጠር-የወንዴ የዘር ህዋስ(ስፐርም) ጉዞን በመግታት የሚደረግ ቀላል ቀዶ ጥገና ነው |  |  |
| 210 | ጡት በማጥባት ለ 6 ወር እርግዝናን መከላከል ይቻላል. |  |  |

**የመልስ ሰጪ የአመለካከት ሁኔታ**

| **ተ.ቁ** | **ጥያቄ** | መልስ | | |
| --- | --- | --- | --- | --- |
|  |  | እስማማለሁ | አልስማማም | አርግጠኛ አደለሁም |
| 212 | የቤተሰብ ምጣኔ የሚጠቀሙ ቤተሰብ ልጆቻቸው የተሻለ ዕድል አላቸው፡፡ |  |  |  |
| 213 | የቤተሰብ ምጣኔ የኑሮ ደረጃን ያሻሸላል፡፡ |  |  |  |
| 214 | የቤተሰብ ምጣኔ እናት ቀጣይ ልጅ ከመውለዶ በፊት እንድጠነክር ይረዳታል፡፡ |  |  |  |
| 215 | ብዙ ልጅ መውለድ ቤተሰብን የደከመና የተሰላቸ ያደርጋል፡፡ |  |  |  |
| 216 | ልጅ መመጠን የልጅ ና አናት ጤናን አይጠብቅም፡፡ |  |  |  |
| 217 | የቤተሰብ ምጣኔ የባል ና ሚስት መተማመን ይቀንሳል፡፡ |  |  |  |
| 218 | ቤተሰብ ምጣኔ የምትጠቀም ሚስት በባላ ትተዋለች፡፡ |  |  |  |
| 219 | የቤተሰብ ምጣኔ የባል ና ሚስት ያጣላል፡፡ |  |  |  |
| 220 | የቤተሰብ ምጣኔ መሀንነት ያመጣል፡፡ |  |  |  |
| 221 | የቤተሰብ ምጣኔ የባል ና ሚስት ደስተኛ ያደርጋል፡፡ |  |  |  |

**ክፍሌ 3፡ ባለቤትሽ ጋር ስለቤተሰብ ምጣኔ አጠቃቀም ስለሚደረግ ውይይት ልጠይቅሽ ነው**

| 301 | ከ**ባለቤትሽ ጋር ስለቤተሰብ ምጣኔ አጠቃቀም ትወያያላችሁ**? | 1. 1.አወ 2.አንወያይም |  |
| --- | --- | --- | --- |
| 302 | ባለቤትሽ ከወሊድ በሑዋላ የወሊድ መቆጣጠሪያ አንድትጠቀሚ ያግዝሻል ? | 1. 1.አወ 2. 2.አያግዘገኝም |  |
| 308 | ከወሉዴ በሁዋሊ ስለሚወሰዴ የቤተሰብ ምጣኔ መረጃ ከየት አገኝሽ; **(**ከአንዴ በሊይ መሌስ መስጠት ይቻሊል**)** | ከጤና ባለሙያ--------------------1  ከጓደኛ -------------------------2 ከመገናኛ ብዙሀን -----------------3  ላሊ ካለ ይጠቀስ ------------------4 |  |

**ክፍል 4 ፡ በእናቶች እና በህፃናት የጤና ክትትል ወቅት ከጤና ባለሙያ ጋር ስለሚደረግ ውይይት እንዱሁም በዛ ወቅት ስለሚኖር የጤና ክትትል ልጠይቅሸ ነው፡፡**

| 401 | ይህ የመጀመሪያሽ ነው? | 1. 1.አወ 2. 2.አይደለም |  |
| --- | --- | --- | --- |
| 402 | በሂወት ስንት ልጆች አሉሽ | ------------ |  |
| 403 | ከዚህ በፊት አስወርዶሽ ያውቃል | 1. 1.አወ 2. 2.የለም |  |
| 404 | የመጨረሻው እርግዝናሽ ሁኔታ እንዴት ነበር ? | 1. የታቀደ 2. ያልታቀደ |  |
| 405 | የመጨረሻው እርግዝናሽ የቅድመ ወሉድ ክትትል ነበረሽ ? | 1.አወ  2.አልነበረኝም |  |
| 406 | ለጥ “406” አወ ከሆነ ፤ ለስንት ግዜ የቅድመ ወሉድ እርግዝና ክትትል አደረግሽ? | 1. 1. አንድ ጊዜ 2. 2. ሁለት ጊዜ 3. 3. ሶስት ጊዜ 4. 4. አራት እና ከዛ በላይ |  |
| 407 | የቅዴመ ወሉዴ ክትትሌ ወቅት ስለ ቤተሰብ ምጣኔ የምክር አገሌግልት አግኝተሻል? | 1. 1.አዎ   2. አላገኘሁም |  |
| 408 | የት ነበረ የወለድሽው ? | 1. 1.በመንግስት ሆስፒታል 2. 2. በጤና ጣቢያ-   3. Health Post   1. 4. የግል ክሊኒክ 2. 5. የግል ሆስፒታል 3. 6. NGO ጤና ተቑም 4. 7. ቤት ውስጥ 5. 8.ሌላ ካለ ይጠቀስ ….. |  |
| 409 | ያዋለደሸ ማን ነበር; | ድክተር--------------------1  አዋሊጅ ነርስ/ነርስ-------------2  ከዘመድቼ አንደ--------------------3 ባሌሰለጠነች ባህሊዊ አዋሊጅ-------4  በሰለጠነች ባህሊዊ አዋሊጅ----------5 ላሊ ካሇ ይጠቀስ---------------------6 |  |
| 410 | የዴህረ ወሉዴ ክትትል ነበረሽ ወይ? | 1. 1.አወ 2. 2.አልነበረኝም |  |
| 411 | በድህረ ወሉዴ ክትትል ወቅትበ ስለ ቤተሰብ ምጣኔ የምክርአገሌግልት አግኝተሻሌ? | 1. 1.አወ   2.አላገኘሁም |  |
| 412 | ወደፊት ልጆች መውለድ ትፈልጊያለሽ ? | 1. 1.አወ 2. 2. አልፈልግም |  |
| 413 | የሚቀጥለውን ልጅሽ መቸ ነው መውለድ የምትፈልጊው? | 1. 1. ከአንድ አመት በሁዋላ 2. 2. ከሁለት አመት በሁዋላ 3. 3.ከሶስት አመት በሁዋላ 4. 4. አራት እና ከዚያ በኋላ |  |
| 414 | ከወሉዴ በሁዋላ የወር አበባሽን ማየት ጀምረሻል? | 1. አዎ  2. አላየሁም | አይዯሇም ከሆነ ወደ ጥያቄ ቁ 503 |
| 415 | \| የወር አበባ ማየት የጀመርሽው መቸ ነው ? \|  \| \| --- \| --- \| | -----------------በሳምንት |  |
| 416 | ከወሉድ በሁዋላ የግብረስጋግንኙነት ማድረግ ጀምረሻል? | 1. 1.አወ   2.አላደረኩም |  |
| 417 | ጥ 416 አወ ከሆነ ; ከወሉድ በሁዋላ የግብረስጋግንኙነት ማድረግ የጀመርሽው መቸ ነው? | 1. 1. ከ 6 ሳምንት በፊት 2. 2.ከ 6 ሳምንት….3 ወር 3. 3. ከ 4……6ወር 4. 4. ከ 7……9 ወር 5. 5. ከ10….12 ወር |  |
| 418 | ከወለድሽ በኁላ ስንት ጊዜሽ ነው | 1. ……………በሳምንት |  |

**ክፍሌ 5፡ ከወሊድ በሁዋላ ስለሚወስድ የቤተሰብ ምጣኔ አጠቃቀም እና ትግበራ ጥያቄዎችን ልጠይቅሽ ነው**

| 501 | ከወለድሽ በሁዋላ ባለው አንድ ዓመት ውስጥ የቤተሰብ ምጣኔ ተጠቅመሻል? | 1. 1.አወ 2. 2.አልተጠከምሁም | ካልተጠከመች ወደ ቁ 505 |
| --- | --- | --- | --- |
| 502 | ከወሊድ በሁዋላ የቤተሰብ ምጣኔ ዘዴን መቼ መጠቀም ጀመርሽ ? | -------በሳምንት ይቀመጥ |  |
| 503 | አወ ከሆነ መልስሽ ; የተጠቀምሽው የቤተሰብ ምጣኔ ዘዴ የትኛው አይነት ነው? | 1. 1. ለአጥቢዎች የሚሰጥ ኪኒን 2. 2. ሉፕ(በማህፀን የሚቀበር) 3. 3. መርፌ- 4. 4.የወንድ ኮንዶም 5. 5. የሴት ኮንዶም 6. 6. በክንድ የሚቀበር 7. 7. የማህፀን ቱቦ ማስቁዋጠር ዘዴ   8. ለ6 ወር የእናት ጡት በማጥባት  9. በ72 ሰዓት ውስጥ የሚወሰድ ኪኒን   1. 10. ሌላ ካለ…. |  |
| 504 | የቤተሰብ ምጣኔን ከየት አገኝሽ;? | 1. 1. ከመንግስት ጤና ተቁዋማት 2. 2. ከግል ጤና ተቁዋማት 3. 3.ከ NGO ጤና ተቁዋማት 4. 4. ከግል ከመዴሀኒት ቤት 5. 5. ሌላ ካለ….. |  |
| 505 | If no ‘Q’ 501 ከወሉድ በሁዋላ የቤተሰብ ምጣኔን ለምን እንዳልተጠቀምሽ ልትነግሪኝ ትችያለሽ? | 1. 1. የጎንዮሽ ጉዳቱን ስለምፈራ 2. 2.በቶሎ እንደገና መውለድ ስለፈለኩኝ 3. 3. የጡት ወተትን መጠን ይቀንሰዋል 4. 4. ከወለድኩኝ በሁዋላ የወር አበባዬን ስላላየሁ 5. 5. ባለቤቴ እንድጠቀም ስለማይፈልግ 6. 6.ስላላገባሁ ( ባል የለኝም) 7. 7. ከወለድኩኝ በሁዋላ የግብረ ስጋ ግንኙነት ስላልጀመርሁ 8. 8. ስለቤተሰብ ምጣኔ በቂ እውቀት ስለሌለኝ 9. 9. ሀይማኖቴ ስለማይፈቅድልኝ 10. 10. .ሌላ ካለ ይጠቀስ… |  |

ስለ ትብብረዎት እጅግ በጣም አመሰግናለሁ፡፡
